# Late-onset doxorubicin-induced congestive heart failure in an elderly cancer survivor: A case report

**DOI:** 10.3389/fcvm.2023.1124276

**Published:** 2023-04-25

**Authors:** Hirotaka Suto, Makiko Suto, Yumiko Inui, Atsuo Okamura

**Affiliations:** ^1^ Department of Medical Oncology, The Cancer Institute Hospital of Japanese Foundation for Cancer Research, Tokyo, Japan; ^2^ Department of Medical Oncology/Hematology, Kakogawa Central City Hospital, Hyogo, Japan; ^3^ Department of Cardiovascular Medicine, Takarazuka City Hospital, Hyogo, Japan

**Keywords:** doxorubicin, cardiotoxicity, onco-cardiology, cancer survivor, elderly, troponin I, global longitudinal strain

## Abstract

**Background:**

Recently, the survival rate of patients with cancer has improved annually due to advancements in cancer diagnosis and treatment technologies. Meanwhile, late-onset complications associated with cancer treatment significantly affect survival and quality of life. However, different from pediatric cancer survivors, there is no unified view on the follow-up of late complications in elderly cancer survivors. We reported a case of congestive heart failure as a late-onset complication of doxorubicin (DXR) in an elderly cancer survivor.

**Case report:**

The patient is an 80-year-old woman with hypertension and chronic renal failure. She received six cycles of chemotherapy for Hodgkin's lymphoma that started in January 201X-2. The total dose of DXR was 300 mg/m^2^, and a transthoracic echocardiogram (TTE) performed in October 201X-2, showed good left ventricular wall motion (LVWM). In April 201X, she suddenly developed dyspnea. Upon arrival at the hospital, a physical examination revealed orthopnea, tachycardia, and leg edema. A chest radiograph showed cardiac enlargement and pleural effusion. A TTE showed diffusely reduced LVWM and a left ventricular ejection fraction in the 20% range. After close examination, the patient was diagnosed with congestive heart failure due to late-onset DXR-induced cardiomyopathy.

**Conclusion:**

Late-onset DXR-induced cardiotoxicity is considered high-risk from 250 mg/m^2^ or higher. Elderly cancer survivors are at higher risk of cardiotoxicity than non-elderly cancer survivors and may require closer follow-up.

## 1. Introduction

Recently, advancements in cancer diagnosis and treatment techniques have improved the survival rate of patients with cancer, including those with Hodgkin's lymphoma, annually (1, 2). On the other hand, late-onset complications associated with cancer treatment significantly affect survival and quality of life (3). In particular, doxorubicin (DXR) cardiotoxicity, which affects survival, is speculated to impair DXR-induced mitochondrial function, ultimately leading to cardiomyocyte death, and correlates with cumulative DXR dosage (4). The incidence of DXR-induced congestive heart failure is as high as 16% when the cumulative dose of DXR exceeds 500 mg/m^2^ and is a critical problem for patients receiving DXR (5). Therefore, the Japanese DXR's package insert includes a dose limit of up to 500 mg/m^2^ of cumulative dosage. However, since a history of radiation therapy to the heart and long-term survival after DXR administration are risks for the development of DXR cardiotoxicity, the European Society of Cardiology guidelines recommend risk-based monitoring such as echocardiography and troponin I (TnI) for pediatric cancer survivors with risk factors, even if the cumulative dose of DXR is lower than 500 mg/m^2^ (6–8). Furthermore, the efficacy of dexrazoxane as prophylaxis for DXR cardiotoxicity has been reported, but effective treatment for DXR cardiotoxicity remains unclear (9, 10). Thus, late-onset complications are considered a problem in oncology for children, adolescents and young adults, and adults, and guidelines have been developed for cancer survivors, including methods for long-term follow-up (6, 11–14). However, there is no unified view on late complications in elderly cancer survivors, who are at risk of cardiac complications, and most of the survivors remain unrecognized. In this study, we reported a case of congestive heart failure as a late-onset complication of DXR in an elderly Hodgkin's lymphoma survivor.

## 2. Case description

**Figure 1:**
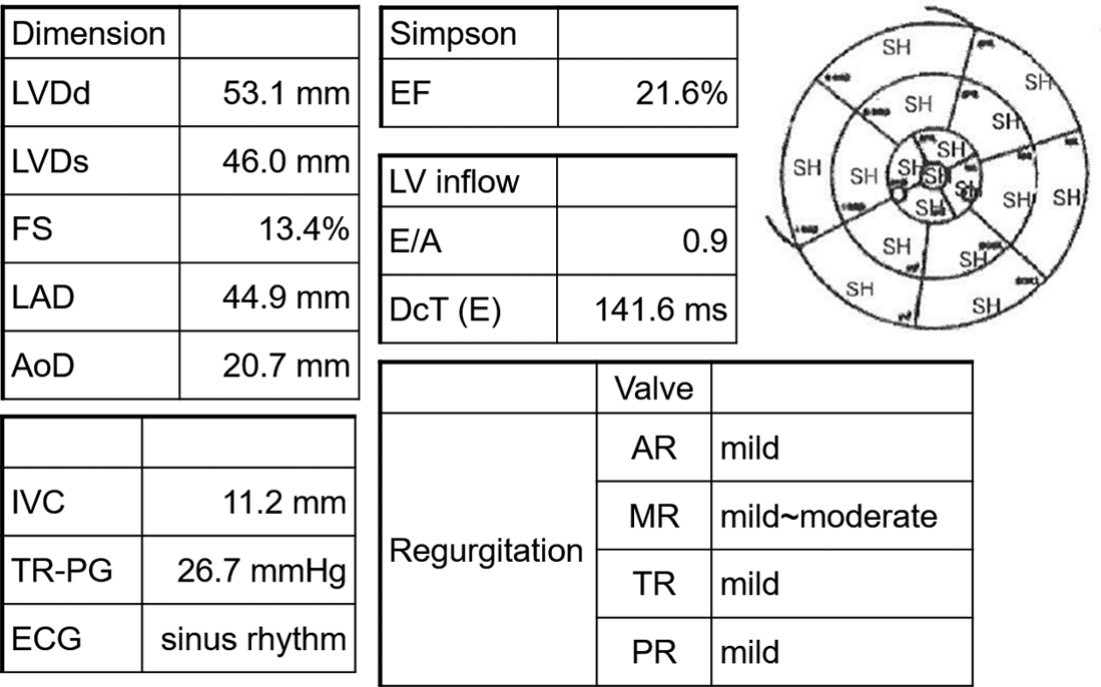
Transthoracic echocardiogram findings at the emergency department visit. LVDd, left ventricular internal dimension in diastole; LVDs, left ventricular internal dimensions in systole; FS, fractional shortening; LAD, left atrial dimension; AoD, aortic root diameter; IVC, inferior vena cava; TR-PG, tricuspid regurgitation peak gradient; ECG, electrocardiogram; EF, ejection fraction; LV, left ventricle; E/A, early-to-late transmitral velocity; DcT, deceleration time; AR, aortic regurgitation; MR, mitral regurgitation; TR, tricuspid regurgitation; PR, pulmonary regurgitation; SH, severe hypokinesis.

**Figure 2:**
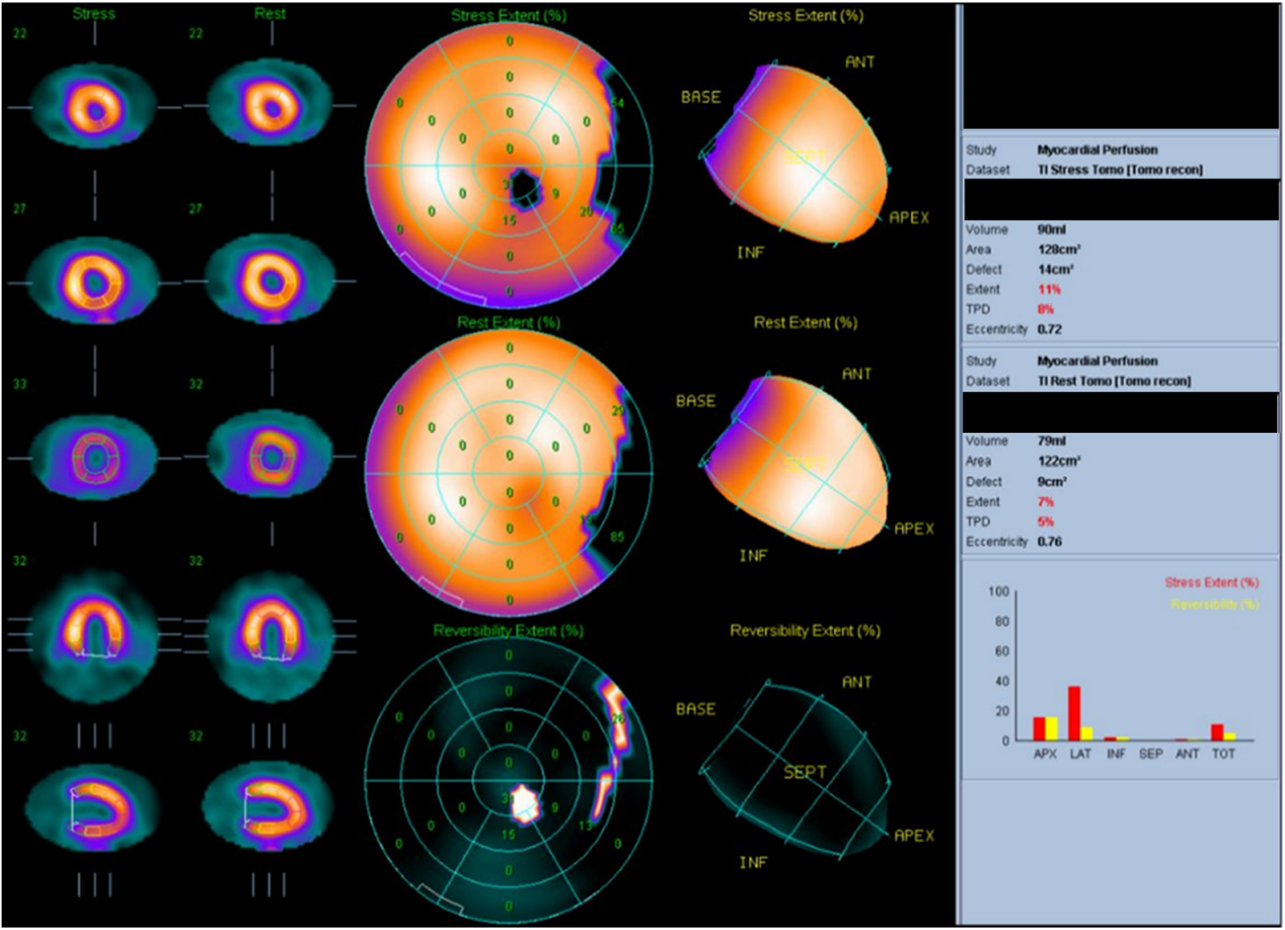
Thallium-201 myocardial perfusion: transient ischemic dilatation and old myocardial infarction of the left circumflex artery.

The patient was an 80-year-old woman with coexisting hypertension, dyslipidemia, and chronic renal failure (CRF). She routinely took enalapril 5 mg once per day and atorvastatin 10 mg once per day. She had no smoking history and no family history of cardiac disease. A transthoracic echocardiogram (TTE) performed in December 201X-3, showed a left ventricular (LV) ejection fraction (LVEF) of 63.4%. She received six cycles of DXR, bleomycin, vinblastine, and dacarbazine (ABVD) for Hodgkin's lymphoma (Lugano classification, stage III) that started in January 201X-2. The total dose of DXR was 300 mg/m^2^, and a TTE performed on October, 201X-2, showed good LV wall motion (LVEF, 65.4%) and no evidence of heart failure. Subsequently, the patient did not experience any recurrence of Hodgkin's lymphoma. In April 201X, the patient suddenly developed dyspnea and was rushed to the emergency room. Upon arrival at the hospital, a physical examination revealed the following: body temperature, 36.9°C; heart rate, 144 beats/min; blood pressure, 160/114 mmHg; respiratory rate, 25 breaths/minute; and oxygen saturation, 98% (under 5 L of oxygen administration). The patient's consciousness was also impaired [Glasgow Coma Scale score, 13 (E3V4M6); Japan Coma Scale score, 10], and her eyelid and conjunctiva were pale. The patient had no heart murmur and no chest pain, but she had mild wheezing, orthopnea, and bilateral leg pitting edema. Blood test results (Table 1) revealed markedly elevated B-type natriuretic peptide (BNP) (1,155 pg/ml) level and slightly increased TnI level (0.08 ng/ml). The patient's hemoglobin (Hb) A1c, free thyroxine, and D-dimer levels were within the normal ranges. The patient also showed decreased Hb level (8.7 g/dl) and increased serum creatinine level (1.48 mg/dl), but these two values had not changed over time for more than a year. An electrocardiogram (ECG) showed sinus tachycardia (Supplementary Figure S1). A chest radiograph showed an enlarged heart and pleural effusion. A TTE showed a diffusely reduced LV wall motion and an LVEF of 21.6% (Figure 1). The patient was diagnosed with clinical scenario 1 acute heart failure (15) and underwent close examination and treatment in the intensive care unit. Thallium-201 myocardial perfusion scintigraphy showed transient ischemic dilatation and old myocardial infarction (OMI) of the left circumflex artery. LV perfusion, LV wall motion, and LV end-diastolic volume results were consistent with tachycardia-induced cardiomyopathy (Figure 2, Supplementary Figure S2). No chest symptoms or significant ECG changes appeared at the time of loading. Since the patient had no evidence of progressive anemia, exacerbation of chronic renal failure, arrhythmia, or acute coronary syndrome and the cumulative dose of DXR was 300 mg/m^2^, the patient was diagnosed with congestive heart failure due to late-onset DXR-induced cardiomyopathy.

**Table 1:** Patient's laboratory results at the emergency room.

Blood components	Patient		Normal range
**Complete blood count**			
White blood cell	11,440	/µl	3,300–8,600
Red blood cell	305 × 10^4^	/μl	386–492 × 10^4^
Hemoglobin	8.7	g/dl	11.6–14.8
Hematocrit	29.3	%	35.1–44.4
Mean corpuscular volume	96.0	fL	83.6–98.2
Platelet	37.4 × 10^4^	/μl	158–348 × 10^4^
Neutrophil	49.9	%	40.0–70.0
Lymphocyte	43.2	%	20.0–50.0
Monocyte	4.7	%	0.0–10.0
Eosinocyte	1.9	%	1.0–5.0
Basocyte	0.3	%	0.0–1.0
**Coagulation test**			
Activated partial thromboplastin time	30.9	sec	26.0–38.0
Prothrombin time	90.0	%	70.0–130.0
D-dimer	0.9	μg/ml	0.0–1.0
**Biochemistry**			
Total protein	7.3	g/dl	6.6–8.1
Albumin	3.6	g/dl	4.1–5.1
C-reactive protein	0.80	mg/dl	0.00–0.14
Aspartate aminotransferase	34	IU/L	13–30
Alanine aminotransferase	14	IU/L	7–23
Alkaline phosphatase	444	IU/L	106–322
Total bilirubin	0.5	mg/dl	0.4–1.5
Lactate dehydrogenase	205	IU/L	124–222
Blood urea nitrogen	28.9	mg/dl	8.0–20.0
Creatinine	1.48	mg/dl	0.46–0.79
Na (sodium)	139	mEq/L	138–145
K (potassium)	5.3	mEq/L	3.6–4.8
Cl (chlorine)	106	mEq/L	101–108
Creatine kinase	59	IU/L	41–153
Creatine kinase-myocardial band	4	IU/L	0–25
Amylase	81	IU/L	44–132
Glucose	216	mg/dl	73–109
Hemoglobin A1c	5.2	%	4.9–6.0
Troponin I	0.08	ng/ml	0.00–0.014
B-type natriuretic peptide	1,155	pg/ml	0.0–18.4
Thyroid-stimulating hormone	5.71	μIU/ml	0.34–4.22
Free thyroxine	1.38	ng/dl	0.77–1.59
**Arterial blood gas analysis** **(under 5 L of oxygen administration)**			
pH	7.351		7.35–7.45
pO_2_ (partial pressure of oxygen)	284.4	mmHg	80–100
pCO_2_ (partial pressure of carbon dioxide)	38.6	mmHg	35.0–45.0
sO_2_ (oxygen saturation)	99.4	%	95–99
HCO_3_^−^ (bicarbonate)	20.9	mmol/L	22–26
Lactate	3.2	mmol/L	0.4–1.8
Base excess	–4.3	mmol/L	–3–3

## 3. Clinical course of treatment

**Figure 3:**
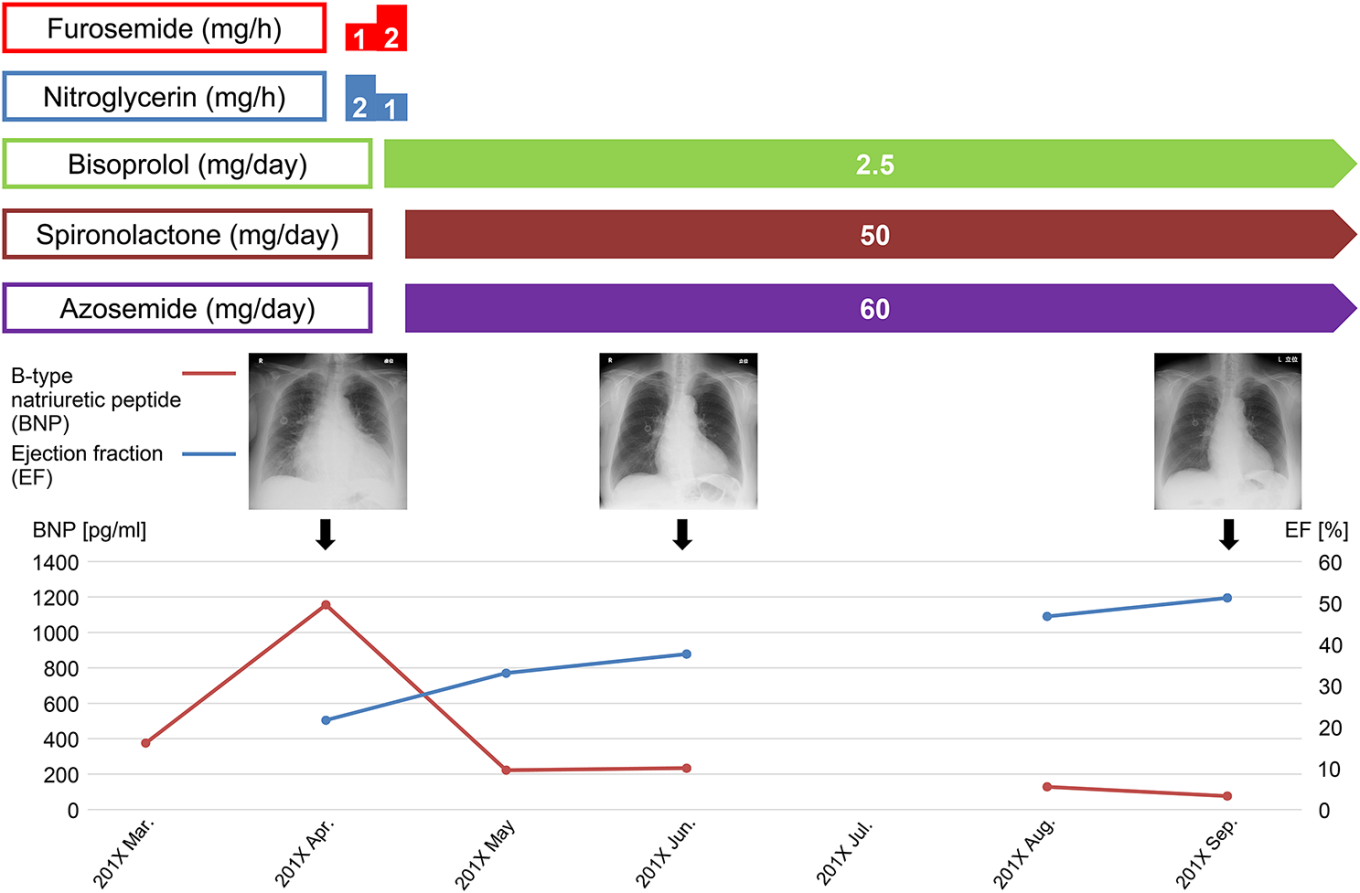
The clinical course of congestive heart failure.

We administered furosemide (1 mg/h) and nitroglycerin (2 mg/h) in the hyperacute phase and added bisoprolol (2.5 mg/day) in the early phase. After the patient's congestive heart failure improved, we continued bisoprolol and added spironolactone (50 mg/day) and azosemide (60 mg/day). After treatment, symptoms, such as leg edema, tachycardia, and orthopnea, disappeared, BNP level decreased to 75.7 pg/ml, and the patient's LVEF improved to 51.2% on TTE in September 201X (Figure 3).

## 4. Discussion

We presented a case of an elderly cancer survivor with late-onset DXR-induced heart failure, whose cardiac function improved with therapeutic intervention.

DXR is a key drug in Hodgkin's and non-Hodgkin's lymphomas and breast and endometrial cancers (16–19). A previous study demonstrated that the 6-year overall survival rate for previously untreated patients with stage III or IV classic Hodgkin lymphoma was 89.4% in the ABVD group (16). Another study reported a 5-year survival rate of 86%–90% for adjuvant chemotherapy containing DXR for recurrent high-risk breast cancer (18).

Pediatric cancer survivors who receive anthracyclines, including DXR, have a persistent risk of developing cardiovascular disease over time. However, the risk of cancer recurrence decreases over time (20, 21). This trend is also evident in adult patients with Hodgkin's lymphoma and breast cancer (22). Patients with breast cancer, even the elderly, reported more deaths from cardiovascular disease than breast cancer 10 years after diagnosis (23).

One potential mechanism for this persistent DXR cardiotoxicity is that DXR accumulates in the mitochondria of cardiomyocytes (24). Accumulated DXR increases the concentration of iron in the mitochondria and produces reactive oxygen species (ROS). ROS may cause myocardial damage, which is a possible reason why the iron chelator dexrazoxane is effective in protecting against DXR-induced myocardial damage (9, 25). Furthermore, topoisomerase IIβ expressed in the myocardium is a key molecular mediator of DXR-induced cardiotoxicity and is thought to impair myocardial repair and irreversibly reduce cardiac function (26–28). Other mechanisms associated with DXR cardiotoxicity include calcium hemostasis imbalance inducing DNA damage and disruption of the neuroglial/ErbB signaling pathway resulting in apoptosis and mitochondrial dysfunction (4). There were recent reports of an association between cardiac syndrome X and DXR-induced endothelial cell damage (29). DXR-induced endothelial cell damage triggers the onset and progression of cardiomyopathy by reducing the release and activity of critical endothelial factors and inducing endothelial cell death. That may be an important mechanism for microvascular angina development in young patients without risk factors or comorbid conditions. The present case was out of the characteristics of DXR-induced cardiac syndrome X due to the presence of OMI, hypertension, and CRF, as well as the patient's advanced age.

DXR-induced cardiotoxicity is dose-dependent, with LV dysfunction occurring in 3%–5% of patients at a cumulative dose of 400 mg/m^2^ compared to 7%–26% at a dose of 550 mg/m^2^ (28, 30). Therefore, the Japanese package insert states that the cumulative dose of DXR should be up to 500 mg/m^2^, but for late cardiotoxicity, DXR has a higher risk starting at 250 mg/m^2^ or higher (6, 31). The risk of cardiovascular disease would be higher in DXR-treated patients aged over 65 years compared to those aged under 65 years (5, 6). In addition, the average life expectancies for Japanese women are 15.6 years at age 75 years and 11.4 years at age 80 years, and follow-up for late complications is necessary even in elderly patients (32). Therefore, elderly patients receiving DXR should be followed up more closely than non-elderly patients, paying attention to late complications. The patient in this case report also received ABVD regimen for Hodgkin's lymphoma at age 78 years, with a cumulative dose of 300 mg/m^2^. Therefore, the risk of late-onset DXR-induced heart failure is high, and close follow-up is necessary.

Although DXR-induced cardiotoxicity is generally considered irreversible, there have been reports of improved cardiac function with early therapeutic interventions with β-blockers, angiotensin-converting enzyme inhibitors, angiotensin receptor blockers, and diuretics (28, 33–35). In the present case, early diuretic and β-blockers administration improved cardiac function with the improvement of sinus tachycardia and reduction of cardiothoracic ratio associated with congestive heart failure (Figure 3, Supplementary Figure S1). Thus, the early diagnosis and treatment of late-onset DXR-induced heart failure may have effectively improved patients' outcomes.

Recently, there have been reports on the prediction of the risk of developing DXR-induced cardiovascular disease based on genes and the value of global longitudinal strain and TnI for the early detection of DXR-induced cardiotoxicity (36, 37).

In conclusion, elderly cancer survivors with an estimated life expectancy of 5–10 years or more are at higher risk of cardiovascular disease and other late complications than non-elderly cancer survivors. Therefore, elderly cancer survivors with a history of DXR treatment may benefit from closer follow-up with BNP and TnI testing and early detection and treatment of DXR-induced heart failure.

## Data Availability

The original contributions presented in the study are included in the article/**Supplementary Material**, further inquiries can be directed to the corresponding authors.
