# Supplementary material for: Late-onset doxorubicin-induced congestive heart failure in an elderly cancer survivor: A case report
Source: Front Cardiovasc Med. 2023 Apr 25;10:1124276. doi: 10.3389/fcvm.2023.1124276 (PMC10166870; doi:10.3389/fcvm.2023.1124276)
Supplement: Supplementary file 1 [file Table1.docx]

Supplementary Material

Late-onset doxorubicin-induced congestive heart failure in an elderly cancer survivor: A case report

Hirotaka Suto^1,2*^, Makiko Suto^3*^, Yumiko Inui^2^, Atsuo Okamura^2^

*** Correspondence:** Hirotaka Suto: [hirotaka.suto@jfcr.or.jp](mailto:hirotaka.suto@jfcr.or.jp), Makiko Suto: maki1129@hotmail.co.jp

# Supplementary Figures


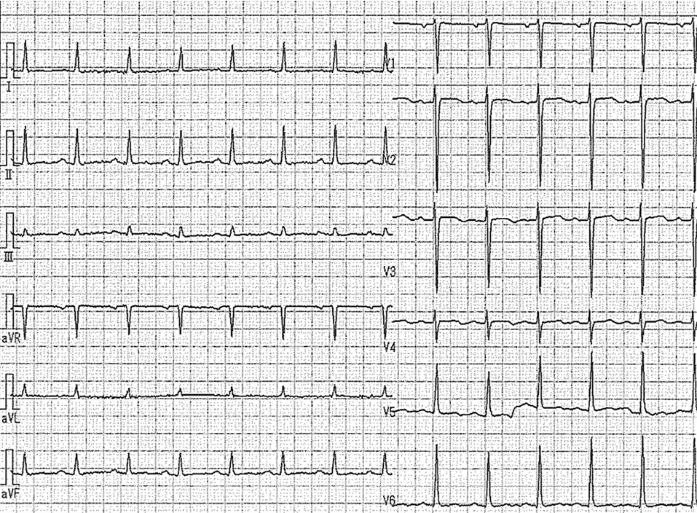

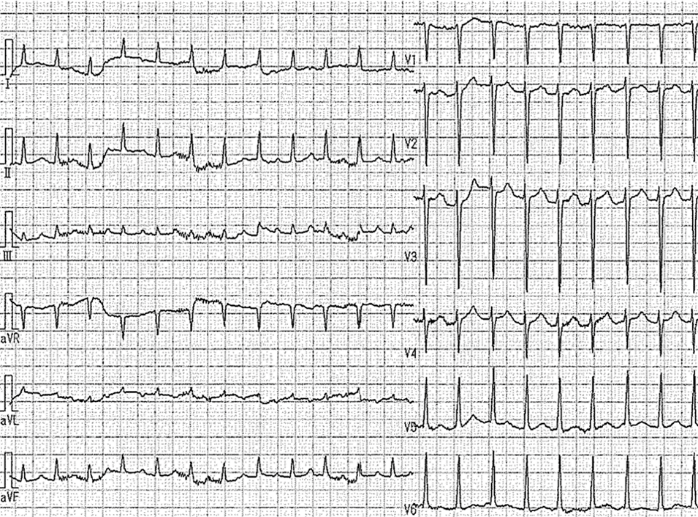


A. ECG on April 6, 201X, at 7:00 pm B. ECG on April 6, 201X, at 7:56 pm

**Supplementary Figure 1.** Electrocardiogram (ECG) findings at the emergency department visit.


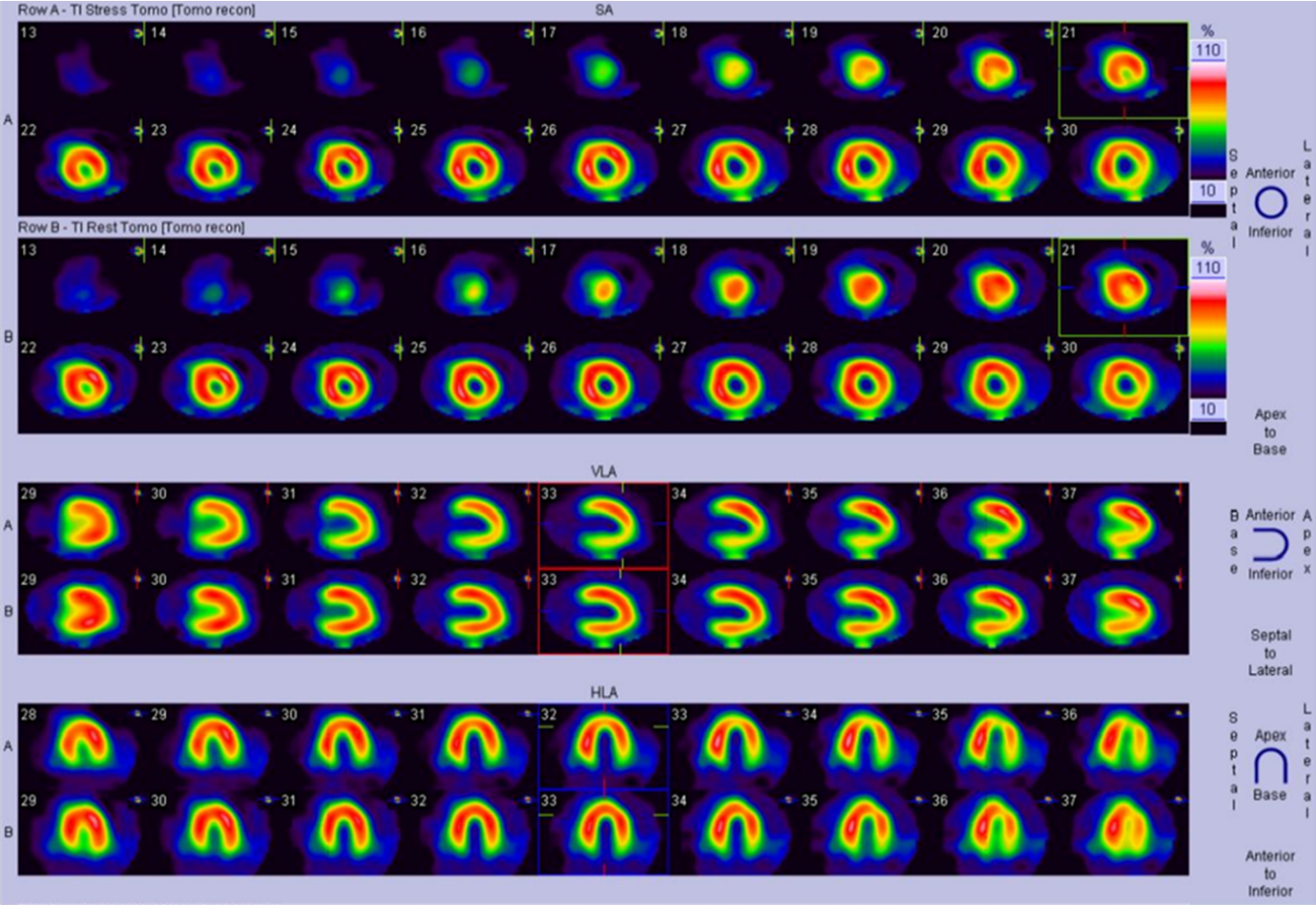


**Supplementary Figure 2.** Thallium-201 myocardial perfusion.

A. Under stress condition

B. At rest
